# Mapping Robots to Therapy and Educational Objectives for Children with Autism Spectrum Disorder

**DOI:** 10.1007/s10803-016-2740-6

**Published:** 2016-02-24

**Authors:** Claire A. G. J. Huijnen, Monique A. S. Lexis, Rianne Jansens, Luc P. de Witte

**Affiliations:** Research Centre Technology in Care, Zuyd University of Applied Sciences, Henri Dunantstraat 2, 6419 PB Heerlen, The Netherlands; Occupational Therapy Department, Zuyd University of Applied Sciences, Heerlen, The Netherlands; Faculty of Health, Medicine and Life Sciences, CAPHRI School for Public Health and Primary Care, Maastricht University, Maastricht, The Netherlands

**Keywords:** Autism spectrum disorder (ASD), Children, Therapy and education objectives, Robots

## Abstract

The aim of this study was to increase knowledge on therapy and educational objectives professionals work on with children with autism spectrum disorder (ASD) and to identify corresponding state of the art robots. Focus group sessions (n = 9) with ASD professionals (n = 53) from nine organisations were carried out to create an objectives overview, followed by a systematic literature study to identify state of the art robots matching these objectives. Professionals identified many ASD objectives (n = 74) in 9 different domains. State of the art robots addressed 24 of these objectives in 8 domains. Robots can potentially be applied to a large scope of objectives for children with ASD. This objectives overview functions as a base to guide development of robot interventions for these children.

## Introduction

An increasing number of children across the globe are being diagnosed with autism spectrum disorder (ASD) (Blaxill 2004; Olds et al. 2013; Scassellati 2005; Wong et al. 2014). From recent studies, a best prevalence estimate of children with ASD of 0.66 % or 1 child in 152 children can be made although also higher numbers have been reported (Volkmar et al. 2014). The Diagnostic and Statistical Manual of Mental Disorders (DSM-V) describes the diagnostic criteria for ASD (American Psychiatric Association 2013). According to the DSM-V, people with ASD often experience persistent problems in social communication and social interaction across multiple contexts on the one hand, and show restricted, repetitive patterns of behaviour, interests, or activities on the other hand. Clinically significant impairments in social, occupational, or other important areas of functioning are apparent (American Psychiatric Association 2013). The symptoms manifest on a continuum, a spectrum, with some individuals showing mild symptoms and others having more severe symptoms and challenges in daily life, and demanding more support (Neurodevelopmental and Group 2012). Together with these differences in severity of symptoms, large variations in symptoms cause ASD to be a highly heterogeneous disorder.

Children with ASD benefit from early and ongoing intervention that is tailored to their specific needs (Volkmar et al. 2014). Even if children reveal progress in some areas during their school time after receiving care, for example in language proficiency, many other areas nevertheless require extensive support, for example in social interaction and communication skills (Volkmar et al. 2014). Most children with ASD continue to have ASD as an adult and continue to experience challenges related to independent living, employment, social relationships and mental health (Myers and Johnson 2007).

Ongoing research has proven the acceptance and efficiency of technology as a support tool for the therapy and education of individuals with ASD and the people who support them on a daily basis (Aresti-Bartolome and Garcia-Zapirain 2014; Boucenna et al. 2014; Goldsmith and LeBlanc 2004; Grynszpan et al. 2014; Lee and Hyun 2015).

Theory of Mind (ToM) refers to the ability to understand one’s own and other people’s beliefs, intentions, desires, imagination, and emotions (Baron-Cohen et al. 1985). Often children with autism have difficulties in ToM. Technologies might provide tools to address these impairments because they can create situations or environments in which children can practice and learn in a safer (e.g. more predictable) and more pleasant manner than when they would practice this (only) with a person. Technologies can deliberately focus on targeting the strengths and weaknesses of the disorder by creating controlled environments that might reduce the anxiety that “real” social situations may cause for children with ASD (Aresti-Bartolome and Garcia-Zapirain 2014). More specifically, socially interactive robots or robot assisted therapy are suggested to be of potential added value in the therapy of children with autism (Cabibihan et al. 2013). Boucenna et al. (2014) suggest a number of reasons for this expected beneficial effect; it might be easier for children with ASD to interact with robots than with humans. Robots (less complex, more predictable, and simpler) can also provide novel sensory stimuli and tend to occupy a special niche between inanimate toys (which do not trigger novel social behaviours for these children) and humans (which can be a source of confusion or even distress for them) (Scassellati et al. 2012). In other words, robots enable embodied interactions that are appealing for children with ASD. Possibly robots can simultaneously provide human-like social cues (e.g. waving, smiling) while maintaining object-like simplicity (e.g. in a consistent manner, limited facial expressions) (Thill et al. 2013). Thill et al. (2013) summarized a number of advantages of using robots for children with ASD: robots can be applied in a controlled manner so that only relevant information is presented minimising the risk of creating stressful and complex situations, robots are better in endless repetition than people, and variations can be made in a conscious (and safe) manner.

Scassellati et al. (2012) report encouraging effects such as increased engagement, increased levels of attention and novel social behaviours, for example joint attention and imitation, when the children interact with robots.

Earlier work (Cabibihan et al. 2013) presented a compilation of robots that have been studied for children with autism and distinguished a number of benefits and roles that robots could have. These roles range from a “friendly playmate”, a “behaviour eliciting agent”, a “social mediator” or a “social actor” to a “personal therapist” (Diehl et al. 2012). A review of the clinical use of robots for individuals with ASDs identified four categories for the roles for interactive robots in clinical applications: the response of individuals (often children) with ASD to robots or robot-like behaviour in comparison to human behaviour, the use of robots to elicit behaviours, the use of robots to model, teach or practice a skill and the use of robots to provide feedback on performance (Aresti-Bartolome and Garcia-Zapirain 2014).

Although most of these studies yielded positive effects using robots for children with autism (e.g. show an increase in desired target behaviours, increased response times, show appreciation/interest for robot interaction), not all children would benefit from (the same) robotic support (Diehl et al. 2012) or would perform better with a human counterpart compared with a robot (Duquette et al. 2008). Mixed results and variability in the nature of the affective response (e.g. positive or negative reaction towards the robot) are also reported; children are not likely to always react positively to the robot (Feil-Seifer and Mataric 2011). This, again, underlines the need for personalised and tailored interventions for this heterogeneous target group.

With respect to teachers’ acceptance on the use of robots in education, one study found that pre-school and elementary teachers accepted a human-like robot to serve as an interactive tool in the teaching process (Fridin and Belokopytov 2014). Other findings regarding attitudes towards the use of robots in (psycho)therapy or education for children show that people, overall, tend to have positive attitudes, considering them as useful and potentially effective tools in psychological treatments or interventions (Costescu and David 2014; Fridin and Belokopytov 2014; Oros et al. 2014).

Despite this work with promising results, the actual current state of application of robots for children with autism in care/therapy and education practices is still relatively in an early stage. More research is needed to understand the actual clinical effects and added value in therapy and education (Diehl et al. 2012). Moreover, it would be interesting to better understand in what areas robots can actually add value to the functioning of children with autism, and how this relates to the “International Classification of Functioning, Disability and Health” (ICF) (World Health Organization 2007). The ICF for children and youth (ICF-CY) provides a classification for health and health-related domains and addresses all aspects of functioning specifically for children and youth.

A critical review by Diehl et al. (2012) concluded that many of these studies are explorative in nature and have methodological limitations and do not necessarily focus on the clinical application of the technology but more on the development of the technology (Diehl et al. 2012). The exploration of robot-based autism intervention has often been directed at clinical or therapy settings and less on educational settings in which children might also benefit from the use of robots in the curriculum (Shamsuddin et al. 2015).

Furthermore, although research has proved the potential added value of different kinds of technologies for children with autism, however, often these tools currently lack the ability to personalise to a specific person’s needs (American Psychiatric Association 2013). Especially for such a diverse and heterogeneous target group as children with autism, it is extremely important that interventions address challenges in different dimensions and a personalised offering is possible (Volkmar et al. 2014). Technologies, including robots might be able to fulfil this requirement as they allow for personalisation and customisation to the individual’s specific needs.

Actual clinical application of robot technology in practice requires the expertise of both technology developers as well as experts in the area of children with ASD. Although public opinion and press devote more and more attention to the use of robots in the therapy or education for children with ASD, scientific peer reviewed publications of systematic clinical effectiveness of the actual implementation of robot based interventions for children with ASD are still scarce.

For robots to be of clinical added value, obviously, teachers and/or care professionals have to accept, adopt and embed these robots in their daily practices. To be used, interventions need to meet the needs of children as well as the needs and practices of these professionals. This is a rather challenging task. For robot developers, it can be quite hard to understand and relate to the needs of this heterogeneous target group and therefore difficult to develop appropriate robot systems to be used as part of interventions. For professionals working with children with ASD on the other hand, the world of social robots seems quite invisible, far away or unreachable. Yet, in order for robot assisted therapy to bring added value to the lives of children with ASD and their carers, connecting professionals from the robotic community with experts in the area of ASD makes a lot of sense.

This study aims to contribute to this by providing a systematic overview of objectives that are important for children with autism and to provide a mapping of available robots to these objectives. This may facilitate the awareness and creation of common understanding between robot developers and ASD professionals (both educators at (special) schools or therapists working in care settings) who are (intending to become) active in the area of robot assisted therapy for children with autism. For ASD professionals it may provide an overview of robots that are currently presented in peer reviewed literature. For the robotic developers on the other hand, it may give insight into relevant ASD domains and objectives that professionals in the field are actually working on.

In short, this research entailed two main goals:

1. To create an overview of relevant therapy and educational objectives that professionals are actually working on in practice for children with ASD.
2. To identify robots focusing on children with ASD that are presented in peer reviewed articles and to relate them to the overview of objectives.

## Methods

A mixed methods approach was used in this study. For the part of creating an overview of ASD objectives that professionals work on for children with ASD, focus group sessions were carried out in which practitioners from the field were involved. For the part of identifying which robots are presented in peer reviewed journals, a systematic literature study was conducted.

### Focus Groups

Care organisations, medical day care centres and special schools, all specialised in supporting children with ASD, were invited to participate in the focus groups to gain insight into the therapy and education objectives professionals work on for children with ASD. At each organisation a session was organised at a moment that was most convenient for the participants from that organisation.

The main principles of the Metaplan method were used for conducting the sessions and the data collection (Schnelle 1979). Main principles of this method include collecting individual input of the participants (one idea on one card), then sharing these in the group in an open non-judgemental brainstorm and ending with organising them collectively.

#### Participants

In total nine focus group sessions were conducted with employees from nine organisations who work with children with ASD on a daily basis. One session was organised for each organisation. This relatively high number of sessions was chosen deliberately in order to be able to identify a large range of objectives inherent to the heterogeneous nature of the disorder and to include both therapy and education settings. The participating organisations all provide care, therapy or education for children and youngsters with ASD (e.g. special need schools, youth care organisations, centres for orthopedagogical treatment, medical day care centres). Professions of the participants ranged from speech-language pathologist, occupational therapist, applied behaviour analyst, game therapist, special needs teacher, psychologist, family coach, to team leader or director.

#### Procedure

For both practical (e.g. busy schedules of care professionals and teachers) and motivational reasons (e.g. increase commitment of professionals), the sessions took place at the premises of the care organisations and/or special schools. The focus groups were carried out in separate sessions (ranging from 4 to 9 participants in each group) at the different locations and took about 2 h each. All participants in one session were employed by the same organisation. Two researchers from the project team were present in each session, one person in the role of focus group moderator, and the other person as preparation assistant, observer, and note taker. As preparation of each session, informed consent papers, post-its and pens were distributed among each participant. To facilitate both the individual and the group aspect, the procedure consisted of 3 main steps. After an introduction, the participants started with listing as many ASD objectives as they considered to be relevant for children with ASD (independently and individually they wrote down one objective per note). The second step was to discuss these individual notes in the group to share results among participants. Finally, all the separate notes with objectives were collectively organised on a large sheet of paper in the middle of the group. For facilitating grouping of the objectives, a categorisation of 12 overall areas was shown as presented in Wong et al. (2014) on evidence based practices for children, youth and young adults with ASD. Participants were free to change, alter or expand these categories where they considered this appropriate. The goal was not to strive for consensus, but to create a realistic overview of the range of objectives that professionals work on with children with ASD. Differences were considered to be valuable, not troublesome.

#### Data Analysis

A picture was taken of the grouping that was done and all notes were collected and digitalised individually. Focus group sessions were recorded (audiotaped, after collecting informed consent) and a transcript was made of each session. The objectives and the clustering that the groups made were collected by two project members who participated in the sessions and they made the overall overview based on these results. An analytical session was organised in which they studied the results and found commonalities or patterns in the mentioned objectives and grouping of the domains. In order to provide a common language for sharing these findings, ICF-CY codes were provided for the objectives. The International Classification of Functioning, Disability and Health (ICF) of the World Health Organisation (WHO) provides a uniform classification of health and health-related domains (World Health Organization 2007). The ICF-CY is the Child and Youth version that is applicable to this study. A member check of the created ASD objectives overview was done by means of an online questionnaire (the participants indicated to agree to the resulting overview) afterwards.

### Systematic Literature Study

#### Procedure

Research articles were obtained through an electronic library search (queried in February 2015) according to the principles stated in the Cochrane Handbook (Higgins and Green 2008). A systematic search was conducted in a number of major databases from various disciplines (ranging from social and behavioural sciences to educational to technology expertise). The consulted databases were: PubMed, CINAHL, EMBASE, ERIC, IEEE Xplore digital library, Science Direct, SpringerLink and Taylor and Francis. Furthermore, a Google Scholar search was performed. For a comprehensive search of the literature, search terms were formulated very broadly to increase the likelihood of inclusion of relevant articles. Three main elements of the search query were used: robot, autism and child. The search terms were tailored to the requirements of the respective databases where necessary (e.g. appropriate use of MeSH terms, headings, thesaurus and free text words). Only articles written in English were included and the search was conducted based on the articles metadata. For more details on the search strategy used in the literature study we refer to the “Appendix”.

#### Data Extraction

All full articles were read by the first author who extracted the following data from these articles: what robot is used in the presented study and for what ASD objective(s) or goal(s) is this robot applied in the specific study? The ASD objectives overview based on the results from the focus groups was used as a framework (see Table 2). For each study, the robot used and the objective that best represents the goals described by the authors was identified. These goals were matched with the objectives in the framework, resulting in a mark in the table.

## Results

### Therapy and Educational Objectives for Children with ASD (From Focus Groups)

#### Descriptive Characteristics

In total, 53 ASD professionals (41 female, 12 male) participated in nine focus group sessions. They were all trained and specialised in working with children with ASD, mostly in multidisciplinary teams with varying backgrounds such as child psychology, psychiatry, behavioural science, speech and language therapy, occupational therapy, physiotherapy, art therapy, special needs education and care or general management. The years of working experience in practice ranged from 1 to 35 years. The large majority of the professionals had an experience of over 5 years (average 12.7 years, SD 7.8 years).

#### Overview of Therapy and Educational Objectives for Children with ASD

During all these sessions, a total number of 489 notes with ASD objectives were created by the participants describing the therapy or educational goals that they consider important for children with ASD. The first two columns of Table 2 present the results from the focus groups and highlight the main areas and objectives that ASD professionals identified as being important goals. The overview is divided into nine main domains; communication, social/interpersonal interactions and relations, self-care/independent living, play, emotional wellbeing, sensory experiences and coping, motor experiences and skills, preschool skills, and functioning in daily reality; each of these domains entail a number of more concrete and specific objectives (linked to ICF-CY codes) (World Health Organization 2007).

Some domains are very closely related, such as communication and social/interpersonal interactions and relations. The objectives within the domains provide more detail of what is meant, and the domain provides the overall context. Participants indicated that all objectives are relevant for children on the spectrum; but not all objectives are urgent for a particular child at any given moment in time. Due to the heterogeneous nature of ASD, the objectives that professionals worked on, differed per child and were dynamic over time. Professionals mentioned that they choose to apply different interventions to work on this variety of objectives. Professionals work with more than one child with ASD, so in their working day at special schools, medical day care centres or ASD care organisations, they are working on multiple objectives using different interventions to achieve their goals. There was a relative equal mix of people working for care organisations providing therapy and professionals working for special needs schools or medical day care centres.

Participants mentioned that a large share of their work is targeted at supporting children to be able to live as independent as possible in different areas of life (e.g. home, school/work, hobby, society). They argued that they focused on improving children’s level of functioning in daily life rather than focusing on the problems they experience.

Tuning of and deciding upon the objectives per individual child is an important task done. Professionals stressed that each child with autism is unique and an enormous variety can be seen between the needs, capacities and challenges of these children. Therefore, they indicated tailoring the objectives to the needs of a particular child at a given time is a crucial task for them. As a result, the range of objectives that professionals worked on differed per individual child and changed over time within each child as well. What works perfectly for one child might lead to a panic attack or discomfort for the other child. Adjusting the detailed and flexible application of interventions to each child is often required to meet the delicate needs of each child. What is a natural reaction for the one child, might seem an almost impossible demand for the other.

### Available Robots (From Literature Study)

**Fig. 1:**
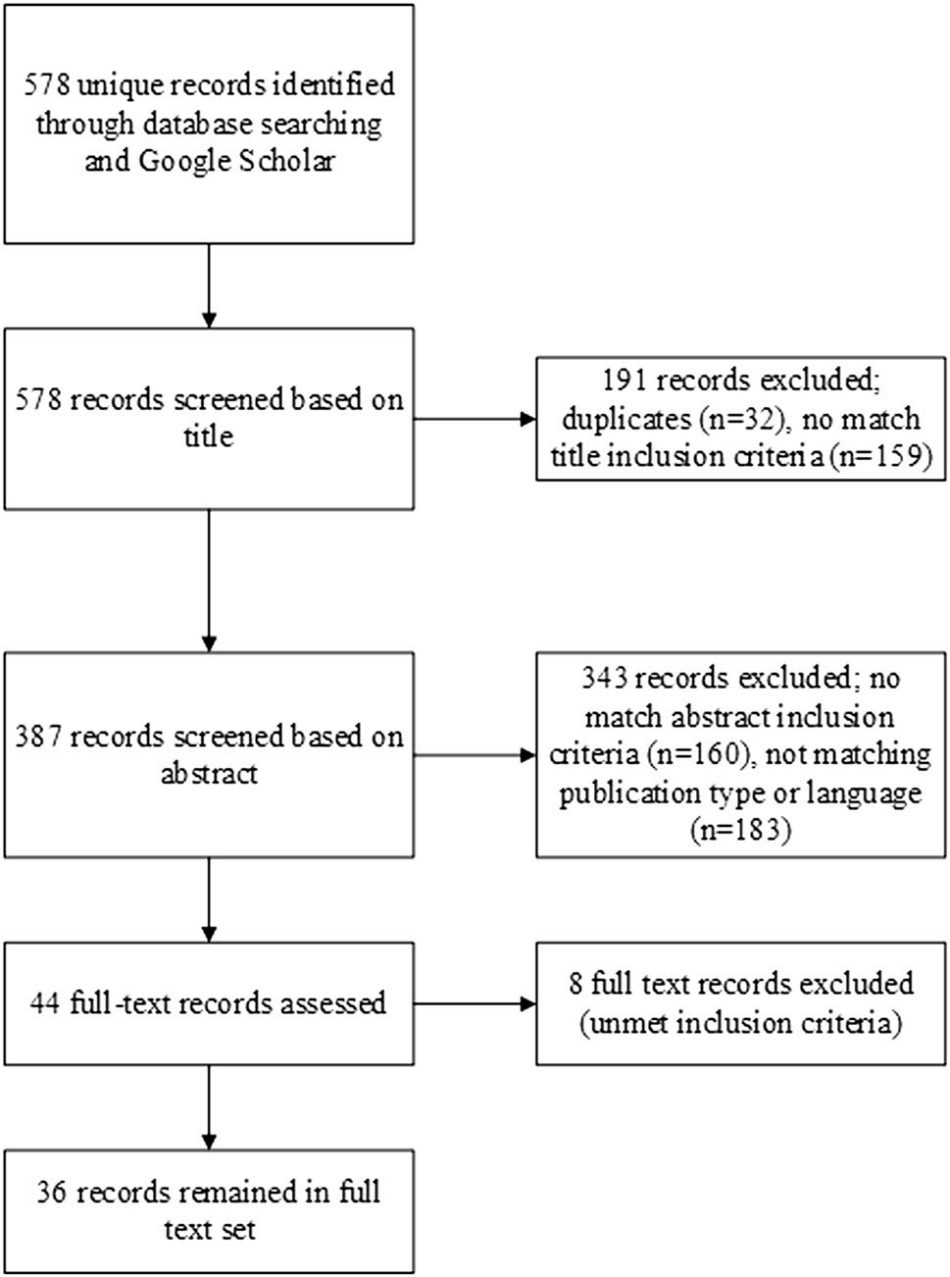
Flowchart of steps in systematic literature search

With all this in mind, we were interested in how robotic support fits in this ASD objectives overview that professionals work with. The initial broad search of the literature search yielded 578 unique references (see Fig. 1 for a visual representation).

Three reviewers from the research team (RvdH, ML, and the first author CH) first screened the titles of these articles according to predetermined inclusion criteria using a 3-point scale (0 = not relevant, 1 = maybe relevant, 2 = relevant). The reviewers were instructed by means of a scoring and inclusion manual. In order to minimise the risk for excluding relevant articles, all references with a minimal score of 2 were included. The second step, abstracts screening (n = 387), was conducted by the same 3 reviewers, again based on a scoring instruction manual. For more details about the inclusion criteria manuals we refer to the “Appendix”. The search resulted in 36 articles that matched our criteria (e.g. robot for children with ASD, tested with children of the target group). Only peer reviewed journal articles were included; book chapters and conference proceedings were excluded.

The reviewers’ Inter-Rater Agreement (weighted Cohen’s kappa coefficient) for scoring the titles and scoring the abstracts between the three reviewers varied between 0.76 and 0.85 (average 0.81).

#### Identifying Robots for Children with an Autism Spectrum Disorder

In total 14 different robots were identified. A number of robots were discussed in multiple articles (e.g. NAO, Robota, Probo, Keepon, Isobot, GIPY-1, KASPAR, and Labo-1), while other robots were identified in one article only (e.g. Cat robot, Tito, HOAP 3, Robot arm, Pleo and Ifbot) (see Table 1).

**Table 1:** Identified robots in peer reviewed journals applied in studies with children with ASD

Robot	Picture^a^	Description	Operating mode	References
Nao		Nao is commercially available, programmable, has multiple degrees of freedom, humanoid robotic platform used in multiple contexts, domains and for varying target groups. More information on Nao can be found on http://www.aldebaran.com/	Autonomous	(Warren et al. 2014)
			Semi-Autonomous	(Warren et al. 2013)
			Controlled/Wizard of Oz	(Tapus et al. 2012)
			Autonomous	(Anzalone et al. 2014)
			Autonomous	(Bekele et al. 2014)
			Controlled/Wizard of Oz	(Huskens et al. 2014)
			Controlled/Wizard of Oz	(Huskens et al. 2013)
			Autonomous	(Bekele et al. 2013)
Robota		Robota is a non-commercially available, multiple degrees of freedom doll-shaped mini-humanoid robot, that was created on the base of a commercially available doll	Controlled/Wizard of Oz	(Billard et al. 2007)
			Controlled/Wizard of Oz	(Robins et al. 2006)
			Controlled/Wizard of Oz	(Robins et al. 2005)
Probo		Probo is developed as multi-disciplinary research platform for human-robot interaction and to develop robot assisted therapies for different children. At the time of writing there are plans for a start-up for Probo. http://probo.vub.ac.be/Probo/buy.htm	Controlled/Wizard of Oz	(Anamaria et al. 2013)
			Controlled/Wizard of Oz	(Vanderborght et al. 2012)
Keepon		Keepon is a commercially available toy robot, designed to study social development by interacting with children, not specifically for ASD. More information at: http://www.mykeepon.com	Controlled/Wizard of Oz	(Kozima et al. 2007)
			Controlled/Wizard of Oz	(Kozima et al. 2009)
			Controlled/Wizard of Oz	(Costescu et al. 2014)
Cat robot		An early model of a robot with cat design features, non-commercially available, developed by a multi-disciplinary researchers group (for children with ASD)	Controlled/Wizard of Oz	(Mun et al. 2014)
I-sobot		I-sobot is a very small commercially available “humanoid” robot: http://www.isobotrobot.com/eng/	Controlled/Wizard of Oz	(Srinivasan et al. 2013)
			Controlled/Wizard of Oz	(Kaur et al. 2013)
Tito		Tito does not seem to be commercially available, it was built using other robot’s existing modular distributed subsystems from https://introlab.3it.usherbrooke.ca/mediawiki-introlab/index.php/CRI	Controlled/Wizard of Oz	(Duquette et al. 2008)
GIPY^b^	^b^	GIPY is a non-commercially available, cylindrical-shaped robot home made by IBISC	Controlled/Wizard of Oz	(Giannopulu and Pradel 2012)
			Controlled/Wizard of Oz	(Giannopulu and Pradel 2010)
Hoap 3		Hoap 3 a programmable Linux robot developed by Fujitsu Automation in Japan that was commercially available. HOAP stands for “Humanoid for Open Architecture Platform”. http://home.comcast.net/~jtechsc/HOAP-3_Spec_Sheet.pdf	Autonomous	(Fujimoto et al. 2011)
KASPAR		KASPAR, a humanoid robot designed by University of Hertfordshire as therapeutic toy for children with autism. Commercialisation plans for KASPAR are in progress. http://www.herts.ac.uk/kaspar/introducing-kaspar/developing-kaspar	Autonomous	(Wainer et al. 2013)
			Semi-Autonomous	(Robins and Dautenhahn 2014)
			Semi-Autonomous	(Costa et al. 2014)
			Autonomous	(Wainer et al. 2014)
Robot arm	not available	A non-commercially available robotic arm model performing a reach-to-grasp action towards a spherical object	Controlled/Wizard-of-Oz	(Pierno et al. 2008)
Pleo		Pleo is a commercially available toy dinosaur robot designed to express emotions and attention, using body movement and vocalization. http://www.pleoworld.com/pleo_rb/eng/index.php	Controlled/Wizard-of-Oz	(Kim et al. 2013)
Labo-1		Robot Labo-1 is a platform with four wheels that drives and turns. http://www.aai.ca/robots/labo1.html	Autonomous	(Dautenhahn 2007)
			Autonomous	(Dautenhahn and Werry 2004)
ifbot		Ifbot robot was used as a prompter for showing different facial expressions	Controlled/Wizard-of-Oz	(Lee et al. 2012)

^a^All pictures are used with permission of the authors

^b^From Giannopulu (2013) and Giannopulu and Watanabe (2016) copied with the permission of the author

One characteristic in which these robots differed was the operation mode, which can vary on a scale ranging from a remote controlled robot (used in many Wizard of Oz studies) to a semi-autonomous robot to a (fully) autonomous robot. Fully autonomous robots (or systems) can act and perform tasks with a high degree of autonomy; without direct input of a person (Bartneck and Forlizzi 2004).

In this case, often, a larger technical environment (e.g. with intelligent sensing camera’s and smart algorithms) is used to observe, analyse and provide input to the robot to act based on a (small) number of pre-programmed tasks. A (remote) controlled robot on the other hand is operated by a person. The operation mode has consequences for the applicability in practice; many differences exists, for example with respect to the technical complexity, infrastructural demands for the use environment, differences in flexibility, price differences as well as different requirements for the people using them.

The operating mode of the presented robots varies between a fully (tele-)operated Wizard of Oz style, to a semi-autonomous or a fully autonomous style. In most of the identified studies, (n = 19, 60 %) the robots are tele-operated and use a kind of Wizard of Oz mode, meaning that a person is (remotely) controlling the robot’s behaviour without the child noticing this. In 31 % of the identified studies, the robots (n = 10) are used in an autonomous manner meaning that no person is controlling the robot, but an autonomous system determines the entire behaviour of the robot. Often an extensive technical and intelligence system is required besides the robot alone to realize a fully autonomous (technical) environment (e.g. sensor input based control logic, vision or camera systems, (head, body parts or eye) tracking devices like a cap to monitor/detect/track child’s behaviour, gazing or even vital signs). In 9 % (3 studies), the robot was used in a semi-autonomous manner, in which part of the robot’s behaviour is triggered autonomously based on the child’s behaviour, and a part of the robot’s actions are tele-operated by a person.

The robot Nao was used in all the three operating modes, in some studies it functioned completely autonomous, in one study semi-autonomous and in others it was tele-operated. Most other robots where most often used in a tele-operation manner except for Robota, HOAP-3, KASPAR and Labo-1 (they were either functioning autonomous or semi-autonomous).

Table 2 shows the overview of identified robots mapped to the ASD objectives overview that was created on the basis of the results of the focus groups. It shows which robots relate to what objectives. Together these 14 robots relate to 24 different objectives out of the total number of 74 ASD objectives identified by the professionals.

**Table 2:**
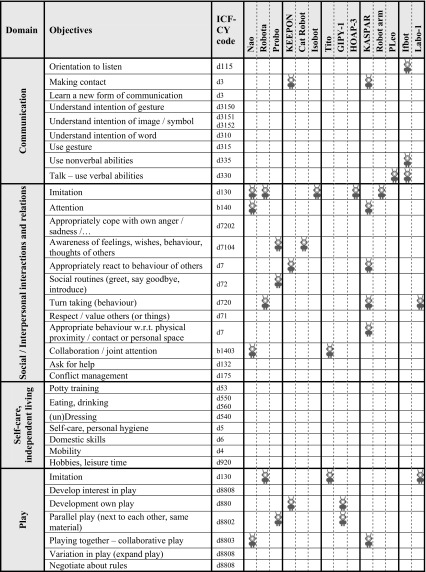

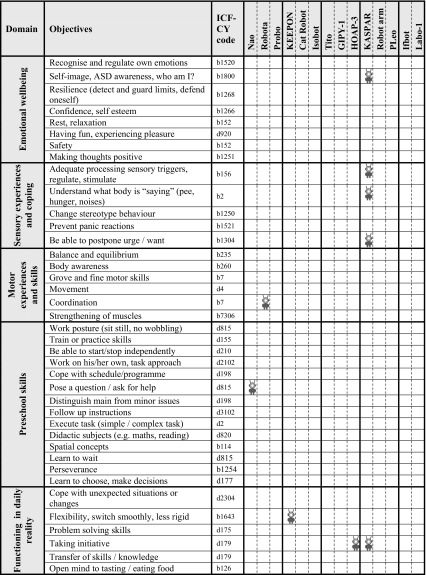
Overview of ASD domains and objectives (results from focus groups) with mapping of robots from literature

Some robots (e.g. NAO, Robota, Probo, Keepon, Isobot, Tito, GIPY-1, KASPAR, Ifbot, Labo-1) have been applied to multiple objectives, and other robots have been reported in the context of one ASD objective only (e.g. cat robot, HOAP 3, Robot arm and Pleo). The Nao robot is discussed in the highest number of different articles (8) and addresses 5 different objectives. KASPAR is presented in 3 articles in the set and is applied to address 12 different objectives.

A number of objectives are targeted by these 14 robots while a rather large number of objectives (n = 50) remain unaddressed by the robots. Objectives that were most often targeted (either presented in more than 2 articles and/or addressed by more than 2 robots) are: imitation (in domain Social/Interpersonal interaction and relations; 7 articles, 5 different robots), turn-taking behaviour (in domain Social/Interpersonal interaction and relations; 5 articles, 3 different robots), imitation (in domain Play; 4 articles, 3 different robots), collaboration/joint attention (in domain Social/Interpersonal interaction and relations; 5 articles, 2 different robots), playing together—collaborative play (in domain Play; 3 articles, 2 different robots), and attention (in domain Social/Interpersonal interaction and relations; 3 articles, 2 different robots).

Table 2 also shows the number of robots that provided support to the different domains. The most commonly addressed domains are: “Social/Interpersonal interactions and relations” (11 robots), “Play” (8 robots) and “Communication” (4 robots). The domain of “Self-care, independent living” is left unaddressed by all robots. “Preschool skills” is the domain where the ASD professionals identified most objectives (n = 14), however, it can be seen that (only) 1 (“pose a question/ask for help”) of these 14 objectives was targeted by 1 robot (Nao) in 1 article. For the domain of “Emotional wellbeing” also 1 robot (KASPAR) could be identified in 1 article addressing 1 objective (“self-image, ASD awareness, who am I”).

## Discussion

The main results of this research indicate that professionals work on a broad variety of therapy and/or educational objectives in a wide range of domains for children with ASD and that state of the art robots focuses on only a small set of these objectives.

The wide range of therapy and educational objectives for children with autism, resulting from the focus groups, is in line with the heterogeneous nature of the disorder (American Psychiatric Association 2013). Professionals indicated that they are focused and driven by supporting these children in coping with their ASD in daily life towards independent living rather than trying to “fix” their impairments, challenges or differences. These objectives could be categorised into 9 domains and 74 objectives.

Best matching ICF-CY codes were collected for each objective (World Health Organization 2007). Since the ICF-CY offers an universal standardised categorisation, it is not specifically constructed for children with ASD. Therefore, in some cases it was challenging to find the best matching ICF-CY code to the objectives, so it was ensured that this task was done with utmost care and attention of multiple project members who were actively involved in the sessions with the professionals.

The participants of the focus group sessions are all highly specialised experts in the area of education or therapy for children with ASD. In the Netherlands many children with ASD attend special schools where they receive special education and dedicated therapy at school. This implies that these professionals are highly specialised in autism, and that the groups of children at schools are rather small (maximum 7–12 children in a classroom) and mostly existing of children with autism. This might be different in other countries and is also changing in the Netherlands (more children with autism will be integrated in regular education).

The results of the literature study, on identifying state of the art robots for this target group, showed that at this moment in time a relatively small subset (n = 24) of this ASD objectives (n = 74) is addressed by the identified robots (n = 14), leaving quite a large number of ASD objectives unmet by robotic support.

Most of the reported studies in this work used a tele-operated Wizard of Oz style in which a person operates the behaviour of the robot. This creates a benefit of flexibility for the human who can sensitively read the social situation and the child and accordingly control the robot to act appropriately. At the same time this also creates a burden (increase of workload) on that person and often extra technical personnel is required to smoothly operate the robot. This is in line with other work stating that few of the current approaches (in robot assisted therapy for children with ASD) use autonomously interactive robots (Thill et al. 2013). Thill et al. (2013) actually call for a need for more autonomous therapeutic robots rather than remote controlled robots.

For a detailed insight into the effects of the robots and types of the studies identified in Table 1, we refer to earlier reviews on the use of robots in the context of ASD (Cabibihan et al. 2013; Diehl et al. 2012). When focusing on the domains, we conclude that the majority of the robot studies were related to 3 of the 9 domains; “Social/Interpersonal interactions and relations”, “Play” and “Communication”. Other domains such as “Self-care, independent living”, “Pre-school skills”, “Emotional wellbeing”, and “Functioning in daily reality” were (largely) unaddressed by the identified robot studies. This is not a surprising result since the main challenges of children with ASD are indeed related to social and communicative challenges as well as impairments in play behaviours (American Psychiatric Association 2013). Typical ASD objectives in these domains, such as imitation, collaborative play, (joint) attention, as well as turn taking behaviour, were often targeted by (quite similar) robotic support in studies. These rather typical ASD objectives are primary difficulties that children with autism experience that in turn create challenges in different areas of their daily living as can be seen in the overview (for example “follow up instructions”). Robotic solutions can possibly also be of surplus value in other (more indirect) areas as well.

When mapping the robotic studies to the objectives overview, we aimed to find the objective in the overview that matches the focus of specific study best.

The overview can function as creating awareness of the scope of objectives for children with autism that professionals are actually working on with children with ASD. The intention is not to suggest to use a robot for all objectives for all children. Developing meaningful robot assisted therapy requires a profound understanding of the target group. To better understand the possibilities and impossibilities, appropriateness or inappropriateness of robotic support in the objectives and domains, more research is needed. For example, using robots to learn children to follow up instructions might be more appropriate than using robots to teach them to negotiate about rules. Moreover, professionals might express a stronger need for additional interventions targeting some objectives rather than others. And some children might react better to interventions using robots than others.

The next step would be that these objectives will be specified and translated into possible robotic interventions that matches the user requirements of both the children and professionals.

As indicated before, especially the diverse and heterogeneous nature of the ASD calls for a high degree of tuning/adaptation/personalisation or individualisation in the interventions. It asks for a bottom-up, client centred, tailor made approach. Robotic interventions might be very well capable of addressing this need due to their many potential advantages, however, current state of the art robots for children with ASD has probably not reached its full potential yet in terms of interventions/clinical application. Furthermore, most of these studies (still) present the robots [operated by a (technical) researcher] as a platform focusing on robot-child interactions rather than a robot assisted intervention in the hands of the care professional embedded into care protocols and actual therapy/educational settings. This is in line with conclusions of earlier work (Bekele et al. 2014; Diehl et al. 2012). This also corresponds with a meta-analysis done on innovative technology based interventions that concluded that no evidence based robot interventions are currently available for children with ASD (Grynszpan et al. 2014). Robot assisted interventions can be seen as a therapy or education tool in the hands of the professionals. In order to be used, these robots do not only have to address the needs of the children with ASD, but they also have to be sensitive to the requirements posed by the professionals. Making it work/happen in practice requires more than the stability and availability of a meaningful robot. If the robot is not incorporated in the care or education provision and application of interventions no child nor professional will ever benefit from robots. In order to do so, we need to better understand the professionals requirements for robot assisted interventions. It is crucial to investigate how robot-based (interaction) scenarios can be integrated into existing therapy/education environments for children with autism (Shamsuddin et al. 2015). Taking this work to the next level implies moving beyond focusing solely on the robot towards embedding a robot in a clinical intervention or therapy/education protocol. For this, more applied research in an education/therapeutic context (e.g. in a school or care setting) is required to understand better what is needed in terms or intervention/education requirements from ASD professionals, the envisioned end-users of robot assisted therapy.

Research has proven the efficacy of many technologies for people with autism. However, although these tools are useful, often these are rather general in nature, resulting in a lack of personalisation to a person’s specific needs (American Psychiatric Association 2013). It is crucial to design appropriate interventions that can be tailored to the individual needs of this target group in order to increase people’s independence and productive functioning (Volkmar et al. 2014).

Technology becomes more and more part of everyday life and activities, and it is inevitable that technology will be integrated into autism intervention as well (McCleery 2015). However, in order to specify and develop meaningful robot based interventions, it is crucial that professionals, stakeholders as well as technology developers co-create (McCleery 2015). This research aimed to provide a the base for understanding relevant objectives in the therapy and/or education of children with ASD, which is a necessary first step in user centred design process for developing robot assisted interventions. In conclusion, this work is expected to be valuable for experts in the area of children with ASD who are considering using robots as innovative tools in education or therapy. Simultaneously, it is considered to be useful for robot developers who are interested in application domains and are in need of a better understanding of the needs of the target group of children with autism.

It may contribute to the creation of common understanding between ASD professionals and robot developers in their (joined) mission to create meaningful robot interventions for children with autism in the quest to support these children to become the best possible version of themselves in life.

## Appendix

### Search Strategy Used in Literature Study

Queries were tailored to the specific databases used, the query for PUBMED for example was

**AUTISM + CHILD + ROBOT**

(((((((((((((((((((((((((((“Child Development Disorders, Pervasive”[Mesh]) OR “Asperger Syndrome”[Mesh]) OR “Autistic Disorder”[Mesh]) OR “pervasive child development disorders”) OR “pervasive development disorders”) OR “pervasive development disorder”) OR “autism spectrum disorders”) OR “autism spectrum disorder”) OR “asperger’s syndrome”) OR “aspergers syndrome”) OR “asperger’s disease”) OR “asperger’s disorder”) OR “aspergers disorder”) OR “asperger disorder”) OR “asperger disease”) OR asperger*) OR “kanner’s syndrome”) OR “kanner syndrome”) OR “kanners syndrome”) OR “infantile autism”) OR “early infantile autism”) OR ASD) OR PDD*) OR PDD-NOS) OR autis*)) AND ((((((“Child”[Mesh]) OR “Child, Preschool”[Mesh]) OR “preschool child”) OR preschool children) OR child*) OR teenager)) AND (((((((“robotics”[Mesh]) OR robotics) OR “humanoid”) OR “non humanoid”) OR “socially assistive robotics”) OR SAR) OR robot* [tiab])

The number of results found per source is displayed in the Table 3.

**Table 3:** Number of references found per source

Source	# of results
CINAHL	20
PUBMED	76
EMBASE	5
ERIC	10
IEEE	175
Science Direct	106
SpringerLink	96
Taylor&Francis	1
Google Scholar	117
Journal Social Robotics	6
Manual search	12
Total references incl duplicates	**623**
Remove bachelor/master thesis	7
Remove duplicates	38
Total unique references	**578**

The bold digits show that these are (sub)totals

### Inclusion Criteria for Scoring Based on Titles Only

- overall question: which *robots* are used (in the therapy or education) for *children* with an *autism* spectrum disorder?
- only titles are provided to minimize the risk for biases (e.g. based on authors or journals)
- English language
- (semantically, not necessarily literally) in title: autism *OR* robots *OR* children
  - we don’t want to restrict too much already only based on the titles. In the next step of scoring abstracts, we will look for autism AND robots AND children. So if in doubt, score 1.
- no medical nor surgical robots
- scorings scores for the titles of the articles
  - 0 = not relevant
  - 1 = maybe relevant
  - 2 = relevant
- All references with a total score of 3 and higher will go to the next step (scoring abstracts)

### Inclusion Criteria for Scoring Based on Abstracts

- English language
- type: journals, conference proceedings, book chapters
- autism + robots + children
  - Which *robots* are used (in the therapy or education) for *children* with an *autism* spectrum disorder?
- no medical or surgical robots
- Scorings scores
  - 0 = not relevant
  - 1 = maybe relevant
  - 2 = relevant
